# Mice with double knockout of H2-Eb1 and H2-Ab1 exhibit reduced susceptibility to allergic rhinitis

**DOI:** 10.1371/journal.pone.0206122

**Published:** 2018-10-29

**Authors:** Zhiyuan Tang, Yan Wang, Liang Lv, Linge Li, Hua Zhang

**Affiliations:** Department of Otorhinolaryngology, the First Affiliated Hospital of Xinjiang Medical University, Urumqi, Xinjiang, P.R. China; South Texas Veterans Health Care System, UNITED STATES

## Abstract

We herein examined the importance of H2-Eb1 and H2-Ab1 in the susceptibility of mice to allergic rhinitis (AR) by developing double-gene (H2-Eb1+H2-Ab1) knockout mice. The mice were randomly grouped into different sensitization and excitation treatments, then their behavioral scores; nasal mucosa HE staining; thymus tissue toluidine blue staining; levels of ovalbumin (OVA)-specific IgE, IL-2 and IL-13 in the serum; and expression of IL-2 and IL-13 in the nasal mucosa were observed. H2-Ab1 and H2-Eb1 were both successfully knocked out in the study group (KO-OVA). Compared with the control group (WT-OVA), the nasal mucosal tissue in the KO-OVA mice showed fewer histological changes, reduced numbers of eosinophilic granulocytes, fewer mast cells in the thymus tissue, reduced concentrations of OVA-specific IgE and IL-13 in the serum, and reduced expression of IL-13 in the nasal mucosa. The behavior of the mice was also improved. In addition, the IL-2 concentration in the serum and IL-2 expression in the nasal mucosa were increased. There were two important findings of this study: (1) The H2-Ab1 and H2-Eb1 double knockout model of allergic rhinitis was successfully constructed, and the Th1/Th2 cell factors were in imbalance in these mice compared to WT mice; (2) the AR susceptibility of the dual knockout mice was reduced, confirming that H2-Ab1 and H2-Eb1 contribute to allergic rhinitis, at least in mice.

## Introduction

Allergic rhinitis (AR) is a type I allergic disease of the nasal mucosa mediated by IgE. Clinically, it is characterized by rhinocnesmus, sneezing, nasal mucus hypersecretion and nasal mucosal swelling [1]. AR is one of the most common diseases encountered in the Departments of Otolaryngology and Head and Neck Surgery. According to conservative estimates, there are more than 500 million patients with AR worldwide [2]. Chronic AR can decrease a patient’s quality of life, lead to financial burdens, and damage the patient’s mental condition. The high incidence of AR means that it has a serious negative impact on public health.

Although the pathogenesis of AR is complicated, an imbalance in T helper (Th) cells and Th factors (the Th1/Th2 ratio) is generally considered to be one of the key findings associated with AR [3]. IL-2 and IL-13 are cellular factors that strongly regulate Th1 and Th2, respectively, and are thus often used as markers of AR [4]. Because studying the pathogenesis of AR in humans is difficult, developing animal models of AR is important to study the disease, and can provide useful information about its pathogenesis and potential targets for prevention or treatment.

Ovalbumin (OVA) is an antigen that has been widely used as a sensitizer in studies of AR [5,6]. In 1990, Japanese scholars used OVA to successfully generate an AR animal model using Brown Norway rats to study anti-allergy drugs [7]. OVA is often used together with an immune adjuvant (such as aluminum hydroxide) to increase its immunogenicity [8]. Aluminum hydroxide can induce a Th2 immune response in mice without significant toxicity [6]. Based on the previous models reported in the literature and our experience [9], we prepared (OVA) with aluminum hydroxide, and the dose was adjusted according to repeated sensitization and excitement principles to sensitize and excite mice to the allergen to induce AR.

The genetic susceptibility to AR has become a hot research topic. For example, Torres-Galvan *et al*. [10] discovered that the HLA-DRB1/DQA1/DQB1 alleles in Spanish people are related to *Dermatophagoides pteronys* sinus allergy. Munthe-kaas *et al*. [11] discovered that HLA-DRBl*13-DQBl*0603 is related to birch allergy, while DQBl*0609-DRBl*13 and DQBl*0501-DRBl*01 are related to Artemisia pollen allergy. Andiappan *et al*. [12] studied the whole genomes of 4,461 Chinese Singaporeans, and found that HLA-DQB1, HLA-DRB1 and HLA-DQA2 are apparently related to AR.

Gene knockout technology uses homologous recombination to displace functional genes with homologous sequences, reducing or preventing the expression of the genes or making the resulting proteins inactive. Using this technology, various genes in the genome of experiment animals can be knocked out to generate new animal models of AR. Kimzey *et al*. [13] used CD28 gene knockout mice and discovered that CD28 blockade prevented respiratory inflammation and high reactivity in an asthma model. Seshasayee *et al*. [14] discovered that blocking OX40L reduced the immune response mediated by thymic stromal lymphopoietin, including Th2 inflammatory cell infiltration, cell factor secretion and IgE synthesis.

According to investigations performed by Cui *et al*. [15,16], DRB1 and DQB1 in the HLA gene family are possible human susceptibility genes for AR. H2-Eb1 and H2-Ab1 are homologous genes of DRB1 and DQB1 in mice [17,18]. Therefore, it was hypothesized that knocking out these genes might be able to prevent or ameliorate the development of AR in mice. In the present study, the Shanghai Biomodel Organism Science & Technology Development Co., Ltd. was entrusted to breed double gene (H2-Eb1+H2-Ab1) knockout mice to generate a mouse model, which was used to assess the importance of H2-Eb1 and H2-Ab1 in AR.

## Materials and methods

### Sources and types of modeled animals

Female mice with different genetic backgrounds were provided by Shanghai Biomodel Organism Science & Technology Development Co., Ltd., including 15 six-week-old SPF C57BIV6 homozygous (H2-Eb1-/- + H2-Ab1-/-) (KO) mice and 30 (H2-Eb1+/+ + H2-Ab1+/+) wild type (WT) mice. All mice were fed in a laminar air-flow isolated feeding room, with a room temperature of 23–26°C, humidity of 40%-60% and a 12 h dark/12 h light schedule. The mouse cages, bedding and drinking water were sterilized under high temperature and high pressure; all mice were fed sterilized fodder to provide whole nutrition for 2 weeks prior to the initiation of any treatment.

### Ethics statement

All animal experiments reported in this study conformed to internationally accepted standards and were reviewed and approved by the Animal Ethics Committee of the First Affiliated Hospital, Xinjiang Medical University (IACUC-20120705003).

### Grouping of studied animals

A random number table was used to assign seven eight-week female KO mice to the study group (KO-OVA) and seven WT mice to the control group (WT-OVA), then the AR model was generated by sensitizing and exciting the mice with ovalbumin. Seven WT mice were administered PBS instead of OVA to generate the blank control group (WT-PBS). The mice were observed for two weeks, and the body weight, growth and behavior were normal in all groups.

### AR modeling in mice

The AR model was developed based on a previously-reported protocol [19]. In brief, during the sensitization phase, 200 μL of ovalbumin and 2 mg of aluminum hydroxide (an immune adjuvant), diluted to a constant volume of 200 μL with PBS, were intraperitoneally injected into each mouse once per day for seven consecutive days. The WT mice in the blank control group were sensitized with PBS solution. During the excitation phase (one week after sensitization), the mice were subjected to atomizing inhalation of 5% ovalbumin solution for 30 min/day for seven days. Then, 10 μL of ovalbumin solution (10% volume) was dripped into the nasal cavities of each mouse every day for seven consecutive days. For the mice in the blank control group, PBS was used instead of ovalbumin.

### Behavioral observation and scoring standards for sensitized and excited mice

Thirty minutes after sensitization, the success of the modeling was judged according to behavior symptom scoring standards [20]. Scores were determined according to the following standards: For nose-scratching (rhinocnesmus), slightly scratching the nose one time corresponded to a score of 1; continuously scratching the nose and face corresponded to a score of 2; scratching everywhere corresponded to a score of 3. For sneezing: sneezing 1 ~ 3 times corresponded to a score of 1; sneezing 4 ~ 10 times corresponded to a score of 2; sneezing more than 11 times corresponded to a score of 3. For hydrorrhea nasalis: nasal mucus flowing to the anterior naris corresponded to a score of 1; nasal mucus flowing to the lower anterior naris corresponded to a score of 2; and a runny nose corresponded to a score of 3. A superposition method was used to obtain total scores (scores for each term were added together for each mouse to obtain 7 scores for each group), a mean score > 5 was considered to indicate that the mouse had AR. According to this scoring standard, the scores of the KO-OVA and WT-OVA groups were both higher than 5, while those of the WT-PBS group were less than 5.

### Evaluations of the nasal mucosal tissues, thymus tissues, and peripheral serum of mice from different groups

Anesthesia (75 mg/kg of ketamine, 5 mg/kg of diazepam, and 0.2 mg/kg of atropine) was induced via intraperitoneal injection in 12-week-old mice with different genotypes. The mice were then euthanized by cervical dislocation, and the nasal mucosal tissues were collected and subjected to HE staining. The gross morphology of the nasal mucosal tissues of mice from different groups was observed and compared under a light microscope. The thymus tissues were also collected and subjected to toluidine blue staining. The numbers of eosinophilic granulocytes in the nasal mucosal tissues and mast cells in the thymus glands were observed and compared by light microscopy. An enzyme-linked immunosorbent assay (ELISA) was used to detect the levels of total IgE, OVA-specific IgE, IL-2 and IL-13 in mouse serum.

### Mouse genotype confirmation by Western blotting and immunohistochemistry (IHC)

Lung tissues were collected and proteins were extracted, then Western blotting was performed to detect the expression levels of the H2-Eb1 and H2-Ab1 proteins in samples from the three groups of mice. IHC was used to detect the expression of H2-Eb1 and H2-Ab1 in the nasal mucosa and thymus samples from the different groups of mice.

### Detection of cytokine expression in the nasal mucosal tissue

Nasal mucosal tissue samples from different groups of mice were collected, then IHC was used to detect the expression levels of IL-2 and IL-13 in the nasal mucosa.

### Statistical analysis

Data are expressed as the means ± standard deviation. The SPSS 17.0 software was used to perform the statistical analyses of the data, and a single-factor analysis of variance was used to determine the significance of differences between groups.

## Results

### Detection of H2-Eb1 and H2-Ab1 expression in mice

**Fig 1:**
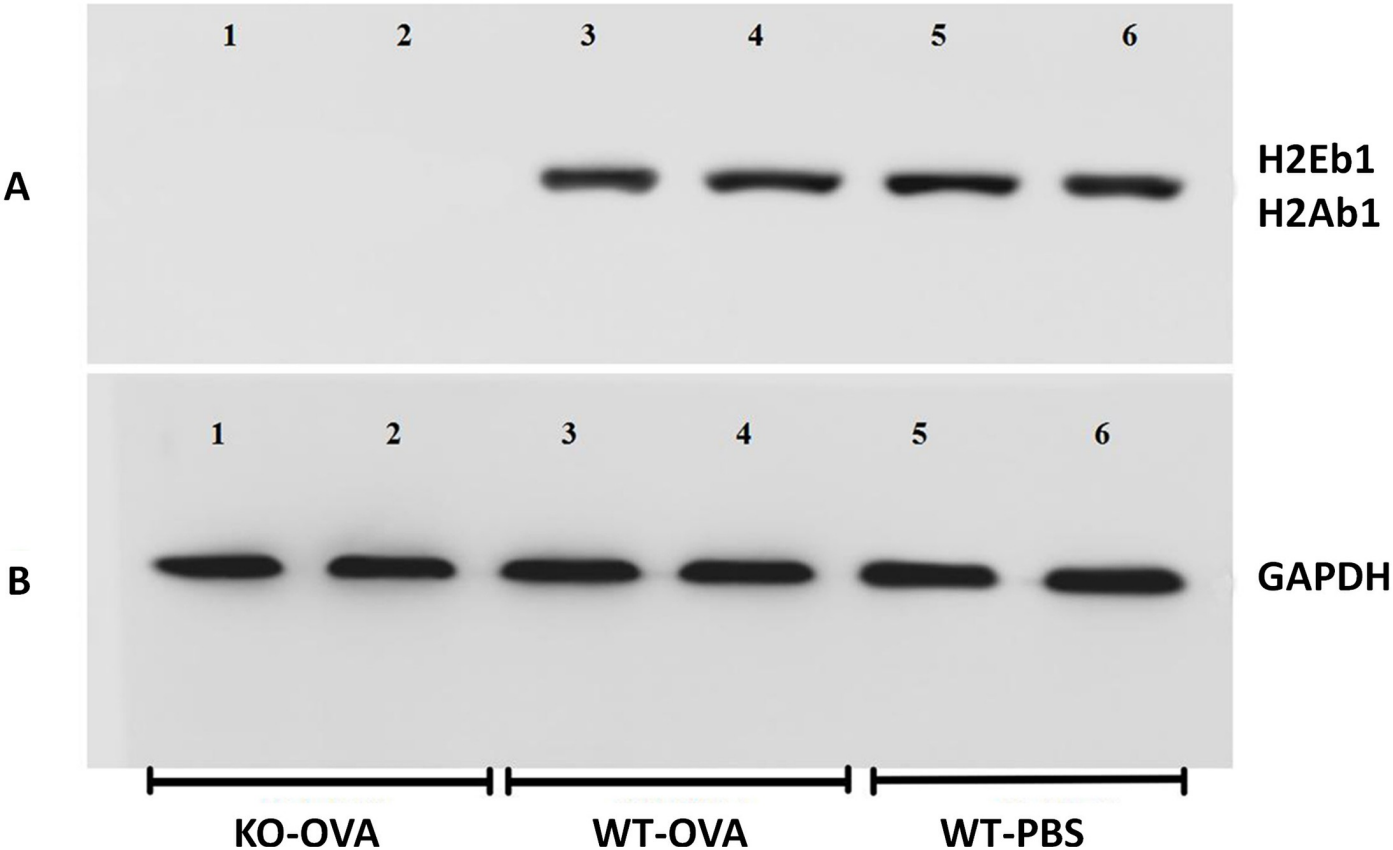
The expression of H2-Eb1 and H2-Ab1 in lung tissue samples from different groups of mice detected by Western blotting. In panel A, the first lane (KO-OVA mice) had no detectable H2-Eb1 protein expression, while the samples in the third lane (WT-OVA mice) and the fifth lane (WT-PBS mice) had H2-Eb1 protein expression. The second lane (KO-OVA mice) also had no detectable H2-Ab1 protein expression, while the samples in the fourth lane (WT-OVA mice) and the sixth lane (WT-PBS mice) had H2-Ab1 protein expression. Panel B shows the expression of GAPDH, which was used as a reference protein.

**Fig 2:**
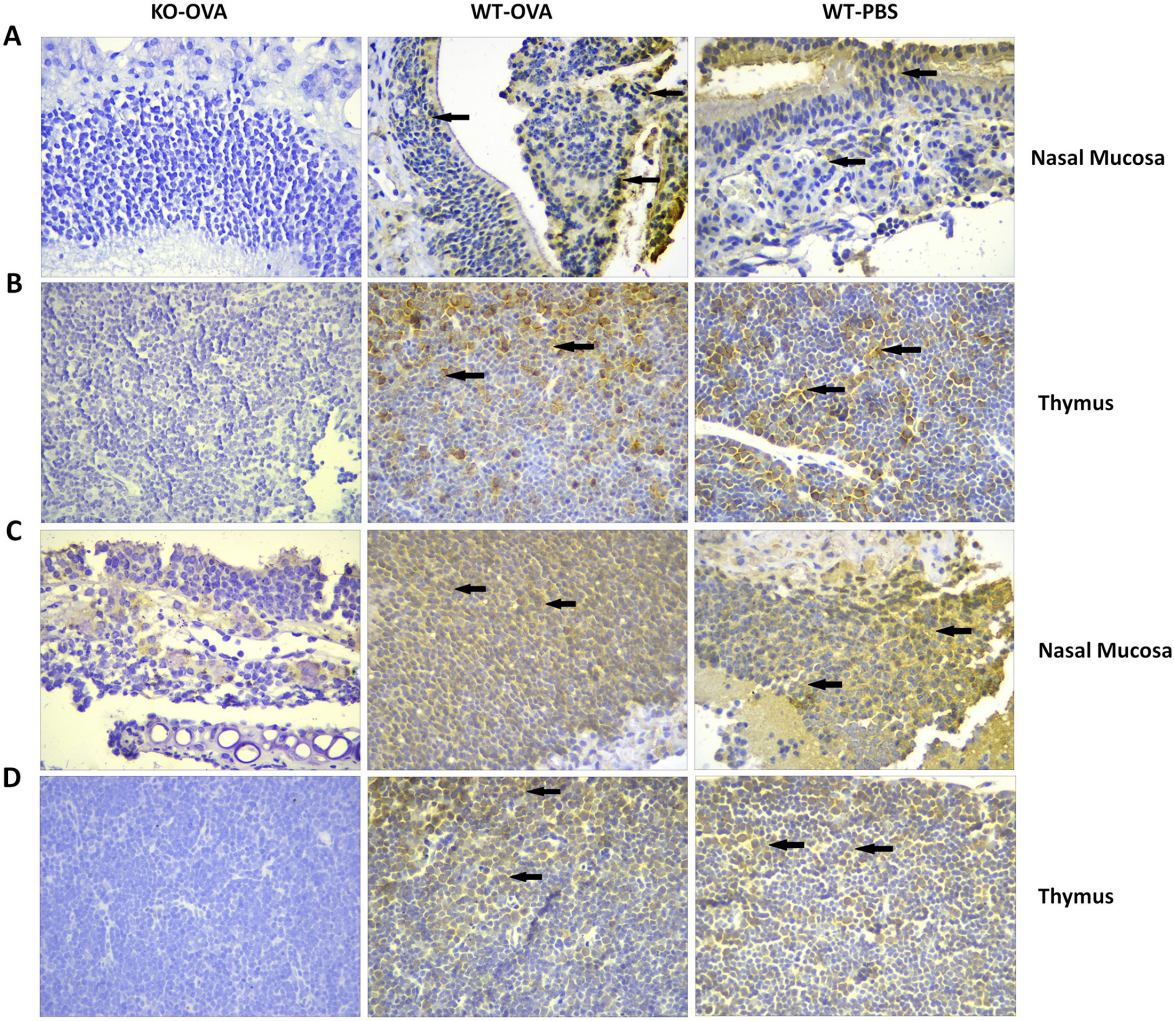
The expression levels of H2-Eb1 and H2-Ab1 in the nasal mucosa and thymus of different groups of mice, as determined by IHC. The panels in A show the H2-Eb1 expression levels in the nasal mucosa for the three groups of mice. The staining of H2-Eb1 in the KO-OVA mice was weakly positive, while there was strong staining in the samples from the other two groups. The panels in B show the H2-Eb1 expression in the thymus, which was similar to that in the nasal mucosa. The panels in C show the H2-Ab1 expression in the nasal mucosa for the different groups of mice. The H2-Ab1 staining in the KO-OVA mice was weakly positive, while the H2-Ab1 staining in the other two groups was strongly positive. Panel D shows the H2-Ab1 expression in the thymus for the three different groups of mice. The H2-Ab1 staining in the KO-OVA mice was weakly positive, while the staining in the WT-OVA and WT-PBS groups was strongly positive.

Western blotting was performed to detect the expression levels of H2-Eb1 and H2-Ab1 in the different groups. The H2-Eb1 and H2-Ab1 proteins in the lung tissue specimens from KO-OVA mice were weakly expressed and not expressed, respectively, while the H2-Eb1 and H2-Ab1 proteins in the lung tissues of WT-OVA and WT-PBS mice were expressed at normal mice (Fig 1). The immunohistochemical staining showed that the proteins were expressed in both the nasal mucosal tissues and thymus tissues of WT-OVA and WT-PBS mice, as indicated by yellow-brown staining. But in the KO-OVA mice, there was no yellow-brown staining (Fig 2), so the results showed that the H2-Ab1 and H2-Eb1 genes in the KO-OVA mice had been knocked out.

### HE staining of the nasal mucosa and toluidine blue staining of the thymus

**Fig 3:**
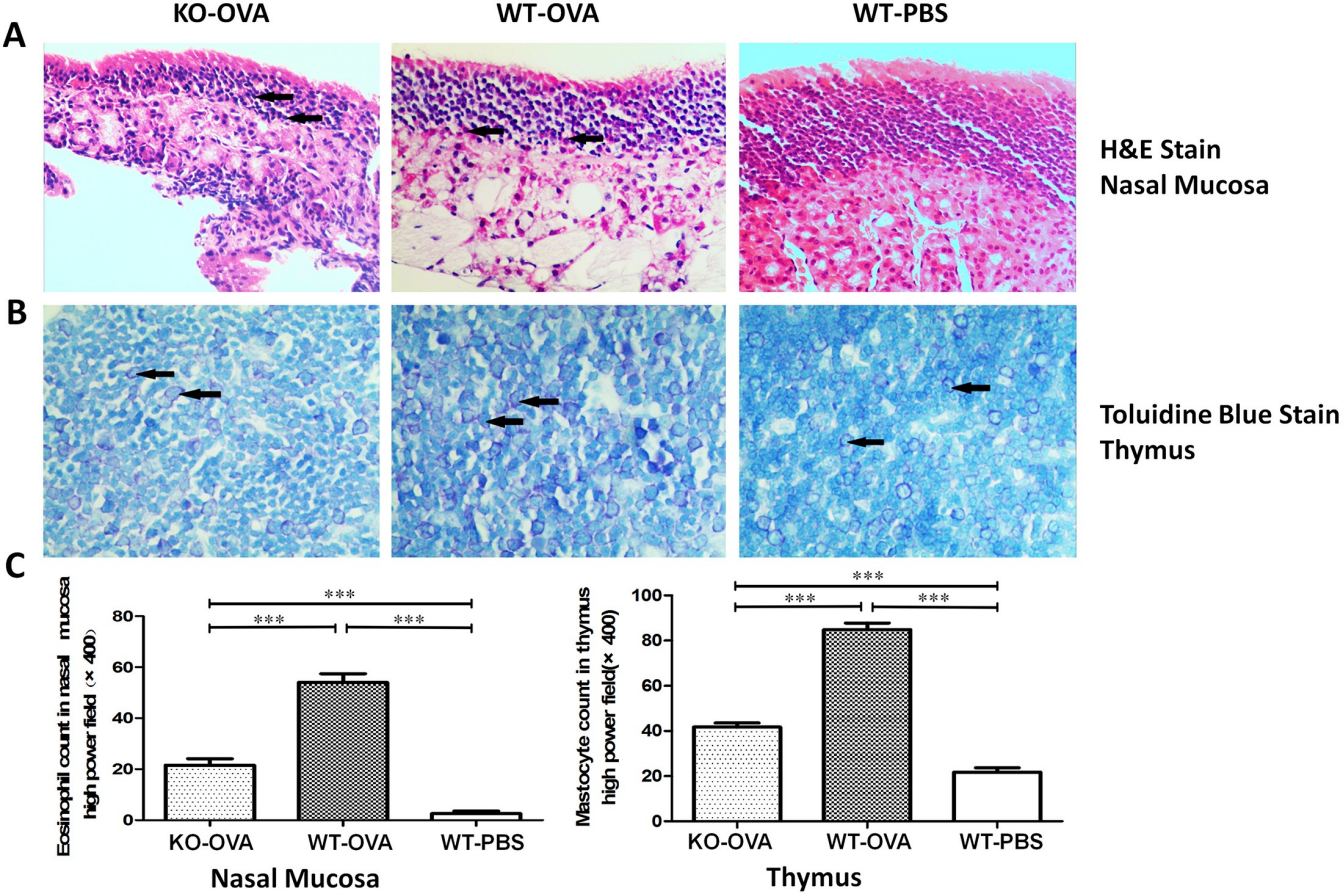
Histology of the nasal mucosal and thymus tissue, and the invasion of eosinophilic granulocytes and mast cells. The panels in A show the H&E (pink) staining of the mouse nasal mucosal (×400) tissues from different groups. The panels in B show the toluidine blue staining of the mouse thymus (×400) samples from different groups. The panels in C show the numbers of eosinophilic granulocytes in the nasal mucosal tissues and mast cells in the thymus tissue (***P<0.001, **P<0.01, *P<0.05). Arrows indicate cells with highly positive staining.

The structure of the nasal mucosal tissue was observed under 400-fold magnification using a light microscope. The KO-OVA mice showed disorder in the mucous blanket on the surface of the nasal mucosa, and the serous gland at the lower mucosa was slightly hyperplastic, with a small number of invaded eosinophilic granulocytes and plasma cells. In the WT-OVA mice, the mucous blanket on the surface of the nasal mucosa was in disorder, some of the mucus was gone, and the serous gland at the lower mucosa was hyperplastic, with a large number of invasive eosinophilic granulocytes and plasma cells. In the WT-PBS mice, the nasal mucosa was complete, the ciliated epithelial cells were arranged in order with scattered goblet cells and sustentacular cells, the basement membrane was even, and the serous fluid and mucous gland at the lower mucosa were evenly distributed. The numbers of eosinophilic granulocytes in the nasal mucosa for the different groups were: 21.57±2.51 in the KO-OVA mice, 54.00±3.12 in the WT-OVA mice, and 2.57±0.98 in the WT-PBS mice. Thymus tissue toluidine blue staining was also observed under 400-fold magnification with a light microscope, and the numbers of mast cells in the thymus tissues for the different groups were: 41.71±1.80 in the KO-OVA group, 84.86±2.97 in the WT-OVA group and 21.71±1.98 in the WT-PBS group. The differences among the groups were statistically significant (***P<0.001, **P<0.01, *P<0.05)(Fig 3).

### Behavioral observations and scoring, the concentration of OVA-specific IgE, and the IL-2 and IL-13 levels in the models

**Fig 4:**
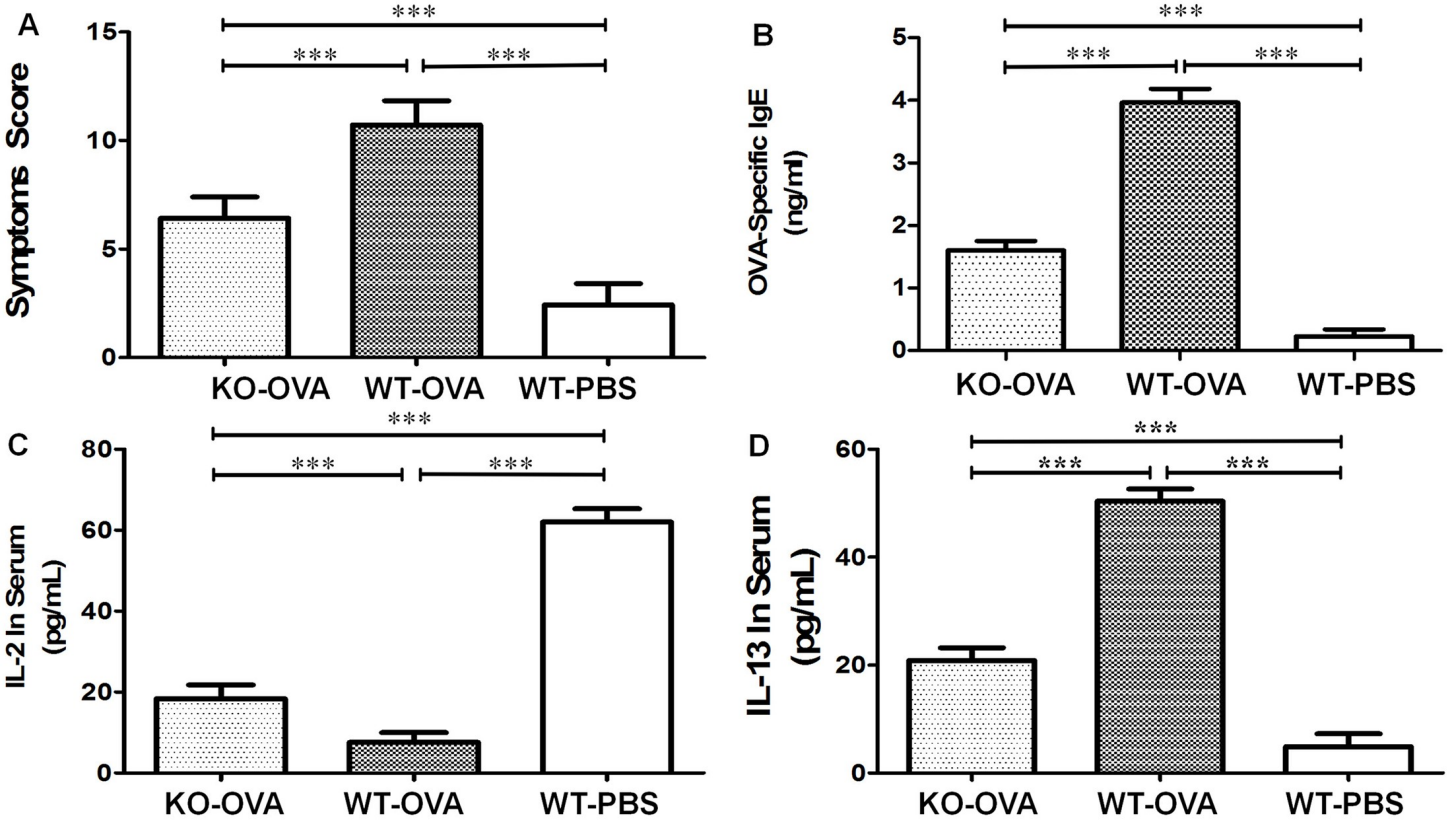
The results of the behavioral observation and scoring, and the concentrations of OVA-specific IgE, IL-2 and IL-13 in serum from the different groups. Panel A shows the behavioral observation scores for the mice in the three different groups. Panel B shows the concentrations of OVA-specific IgE in the serum of mice from different groups. Panel C shows the concentrations of IL-2 in the serum of mice from the different groups. Panel D shows the serum concentrations of IL-13 in the mice from different groups (***P<0.001,**P<0.01,*P<0.05).

After the mice in the KO-OVA and WT-OVA groups were sensitized 3–4 times, nasal scratching was observed, while no scratching was observed in the WT-PBS group. During the final excitation period, scratching, nose swelling, and discontinuous sneezing were observed in the KO-OVA and WT-OVA mice, while the WT-PBS mice did not exhibit any abnormal behaviors (Table 1). The comprehensive score of the WT-PBS group was less than 5, while the scores for the KO-OVA and WT-OVA groups were both greater than 5, with the score for Group WT-OVA being higher than that of Group KO-OVA. The results of an ELISA indicated that the OVA-specific IgE concentrations in the serum from mice in the KO-OVA (1.60±0.15) and WT-OVA (3.96±0.22) groups were higher than those in the WT-PBS group (0.23±0.11). The IL-2 concentration in the serum of mice in the WT-PBS group was increased (62.03±3.27), and was higher than that of the mice in the KO-OVA (18.40±3.37) and WT-OVA (7.60±2.37) groups. Similarly, the IL-13 concentrations in the serum of the KO-OVA (20.87±2.33) and WT-OVA (50.40±2.26) mice were higher than that of the WT-PBS mice (4.92±2.37). The differences among the three groups were statistically significant (Fig 4).

**Table 1:** The average score of allergic rhinitis symptoms in mice ($\bar{\text{x}}\pm \text{s}$).

Group	N	Rhinocnesmus	Sneezing	Running Nose	Comprehensive Score
**KO-OVA**	**7**	**1.86±1.07**	**2.14±0.90**	**1.29±1.38**	**6.43±0.98**
**WT-OVA**	**7**	**3.58±0.79**	**4.14±0.69**	**3.00±0.82**	**10.71±1.11**
**WT-PBS**	**7**	**0.71±0.76**	**0.71±0.49**	**0.86±0.69**	**2.43±0.98**
**F**		**18.612**	**40.875**	**8.859**	**114.727**
**P**		**<0.01**	**<0.01**	**<0.01**	**<0.01**

### Detection of IL-2 and IL-13 in the nasal mucosa by IHC

**Fig 5:**
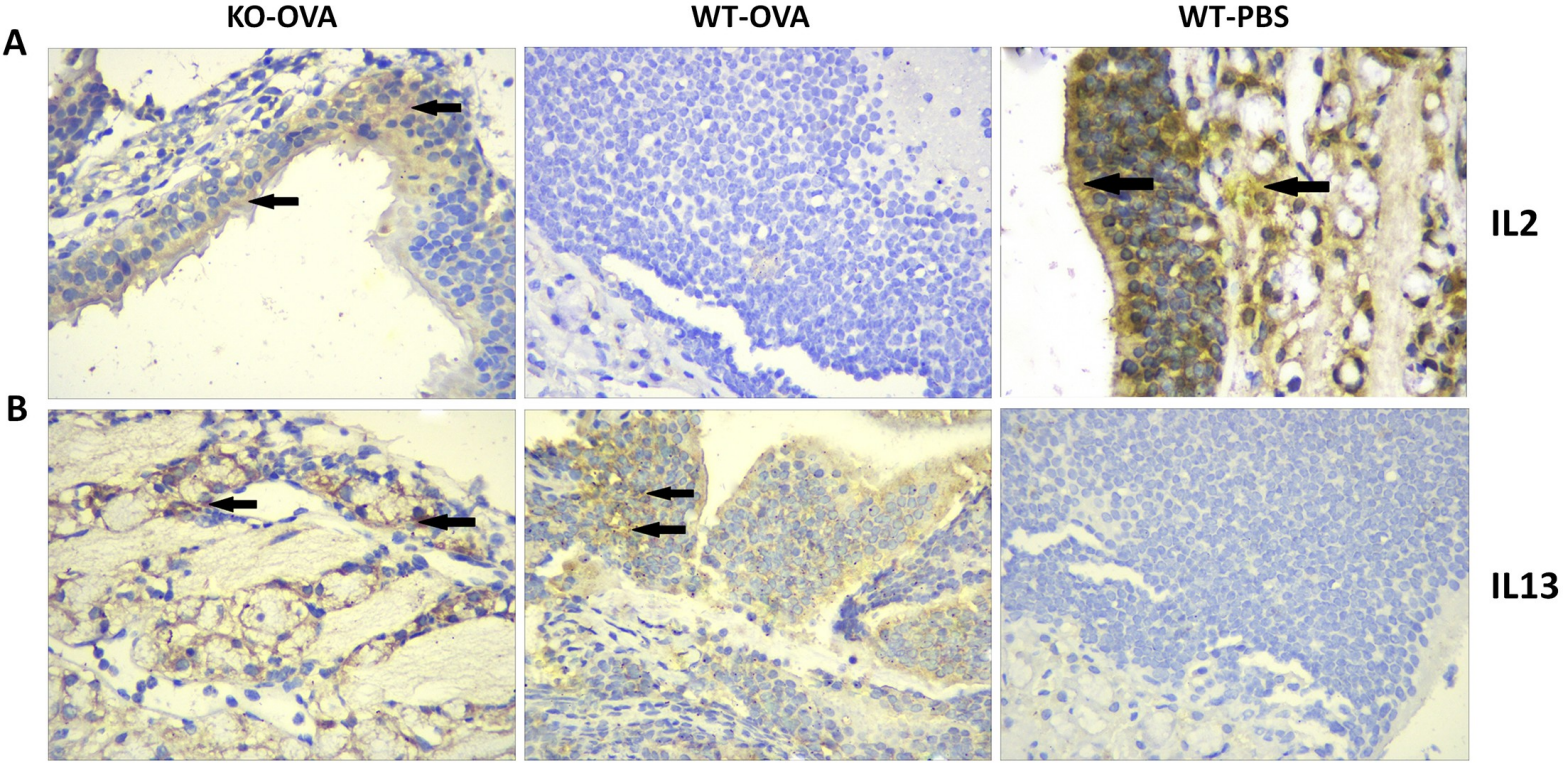
The expression of IL-2 and IL-13 in the nasal mucosa of different groups of mice, as detected by IHC. The panels in A show the IL-2 staining in the different groups, while the panels in B show the IL-13 staining in these groups. The arrows indicate highly positive areas.

The nasal mucosal cytoplasm and capsule were subjected to IHC to assess the expression of IL-2. The WT-PBS mice showed strongly positive (85%) expression of IL-2 (brownish-yellow staining). While the positive cell ratio in the KO-OVA mice (30%) was lower than that in the WT-PBS mice (85%), it higher than that in the WT-OVA mice (2%). Immunohistochemical staining for IL-13 showed that it was expressed in the nasal mucosal tissues of the WT-OVA mice and KO-OVA mice, with only weakly positive staining in the WT-PBS mice (1%). The positive cell ratio in the WT-OVA mice (76%) was higher than that in the KO-OVA mice (25%) (Fig 5).

## Discussion

It is necessary to perform a series of subjective and objective evaluations to judge whether a model has been successfully constructed. In the present study, we used behavioral symptom scoring standards for mice, where a score > 5 indicated that the model was successfully constructed (*i*.*e*., that the mice developed AR) [21]. Although the scores of the KO-OVA and WT-OVA groups were both above 5, indicating that the mice have symptoms of AR, the score of the WT-OVA group was higher than that of the KO-OVA group, suggesting that the AR susceptibility of the KO-OVA mice without H2-Eb1 and H2-Ab1 was reduced.

We also assessed the histological changes in the mice. The nasal mucosal staining showed that the nasal mucosa of both the KO-OVA and WT-OVA mice had been invaded by inflammatory cells and eosinophilic granulocytes, and the tissues were loose and swollen, the submucous gland was hyperplastic, and the mucosal vessels were enlarged. However, the AR-associated pathological changes of the nasal mucosa in the WT-OVA mice were more severe than those in the KO-OVA mice. The thymus tissue toluidine blue staining showed that there was invasion of a large number of mast cells, and numbers of eosinophilic granulocytes and mast cells were counted under a high-power microscope (×400). The findings showed that the number of invaded cells in the WT-OVA mice was higher than that in the KO-OVA mice, which was higher than that in the WT-PBS mice, providing evidence that the AR model was successfully constructed and suggesting that the wild type mice with expression of both genes (H2-Eb1+H2-Ab1) were more susceptible to AR than the mice in which both genes (H2-Eb1+H2-Ab1) were knocked out.

Allergic rhinitis (AR) is a type I allergic disease of the nasal mucosa mediated by IgE. Upon binding to its receptor, IgE activates the mast cells and basophilic granulocytes to release histamine, leukotriene and other mediators, which stimulate the nasal mucosa and result in rhinocnesmus, sneezing, nasal mucus hypersecretion, nasal mucosal swelling and paleness [22]. Therefore, an increase in the specific IgE titer is characteristic of the specific inflammatory response associated with AR, and evaluating the IgE production in models helps to assess their clinical relevance [23,24]. The present study showed that the concentration of OVA-specific IgE in the gene knockout mice was between the WT-OVA and WT-PBS groups, which suggested that the mice without H2-Eb1 and H2-Ab1 were more resistant to AR.

In 1986, Mosmann [25] discovered that mouse cells could be divided into Th1 type and Th2 type cells according to their biological function and mode of generation. In a normal organism, the Th1 and Th2 cells are in relative balance, and an imbalanced Th1/Th2 ratio can result in tumor growth [26,27], autoimmune disease [28,29] and infectious disease [30]. Although the immune system and the propagation of an immune response is complicated and involves a variety of cells, it has been shown that an imbalanced Th1/Th2 immune response or over-differentiation of immune cells into the Th2 phenotype can lead to hyper-responsiveness of the nasal mucosa, resulting in AR [2]. Numerous animal and clinical experiments have verified that, in the setting of AR hypersensitivity, Th2 cells were hyperpolarized and Th2 cell factors, such as IL-4, IL-5 and IL-13, were overexpressed, while IFN-γ, IL-2, and Th1-type cell factors were inhibited [30]. The experiments performed in our newly-constructed AR model using gene knockout mice showed that the IL-2 and IL-13 concentrations in the serum of the KO-OVA and WT-OVA mice were different from those of the WT-PBS mice. Moreover, IHC staining of the nasal mucosa of the three groups of mice demonstrated that the IL-13 expression in the KO-OVA and WT-OVA mice was stronger than that in the WT-PBS mice, while the IL-2 expression in the WT-PBS mice was stronger than that in the KO-OVA and WT-OVA mice. Previous studies have suggested that IL-2 is negatively correlated with the severity of AR, while IL-13 is positively correlated with the severity of AR [31], and this was also shown in our mice. This may suggest that in the wild type mice (WT-OVA, no gene knockout), the Th1/Th2 immune response was more imbalanced than in the homozygous mice (KO-OVA; HA-Eb1 and HA-Ab1 knocked out). Although more work will be needed to assess the functions of these genes, and whether the findings translate to humans, the present study provides a new model for studying AR and may suggest targets for the prevention or treatment of AR.

## Conclusion

In the present study, we developed a clinically-relevant animal model of AR using gene knockout technology. Although this model is still in its initial stage, the present findings suggest that H2-Eb1 and H2-Ab1 are associated with the susceptibility of mice to AR, which lays a foundation for subsequent experiments and provides an additional method and route for exploring the pathogenesis of AR. Further studies comparing mice with knockout of the two individual genes should be performed to determine which gene contributes more strongly to the pathogenesis of AR, and whether their functions are redundant. In addition, the effects of genes on organisms are complicated, and the other effects of H2-Eb1 and H2-Ab1 are currently unknown, and should be explored in subsequent studies.
